# Location for Practice

**Published:** 1869-03-15

**Authors:** 


### *Location for Practice.*

Dr. E. Hollinsworth, of National, Clayton County, Iowa, will sell his residence, (a fine one) and practice, for $2,000. He is the only physician in the place, and the field is a good one.

A favorable opportunity may also be heard from by addressing P. J. Mulvane, M. D., Wyanet, Bureau County, Illinois.

